# PD-1/PD-L1 expression in a series of intracranial germinoma and its association with Foxp3+ and CD8+ infiltrating lymphocytes

**DOI:** 10.1371/journal.pone.0194594

**Published:** 2018-04-04

**Authors:** Bin Liu, Yoshiki Arakawa, Ryuta Yokogawa, Shinya Tokunaga, Yukinori Terada, Daiki Murata, Yasuzumi Matsui, Ko-ichi Fujimoto, Nobuyuki Fukui, Masahiro Tanji, Yohei Mineharu, Sachiko Minamiguchi, Susumu Miyamoto

**Affiliations:** 1 Department of Neurosurgery, Kyoto University Graduate School of Medicine, Kyoto, Japan; 2 Department of Diagnostic Pathology, Kyoto University Graduate School of Medicine, Kyoto, Japan; Université Paris Descartes, FRANCE

## Abstract

One histopathological characteristic of intracranial germinoma is abundant tumor-infiltrating lymphocytes (TILs) showing a two-cell pattern with large undifferentiated tumor cells. The programmed cell death 1 (PD-1)/programmed cell death 1 ligand (PD-L) axis has recently been recognized as an anti-tumor immune system. To evaluate intratumor immune status in intracranial germinoma, we examined expressions of PD-1 and PD-L1 (clone 28–8) and subtypes of TILs. Expressions of PD-1 and PD-L1 were detected immunohistochemically in 25 formalin-fixed, paraffin-embedded tumor specimens from 24 patients with intracranial germinoma consisting of 22 primary and 3 recurrent tumors. To evaluate subtypes of TILs, quantification of lymphocytes with CD3, CD8, CD4, and Foxp3 was performed. Statistical analyses were performed among PD-1, PD-L1 and subtypes of TILs. In 25 tumor tissue, expressions of PD-1 in TILs and PD-L1 in tumor cells were identified in 96% (24/25) and 92% (23/25), respectively. Expression of PD-1 was associated with CD3+ TIL density. Expression of PD-1 correlated with Foxp3+ TIL density and CD8+ TIL density, but not with CD4+ TIL density. Furthermore, expression of PD-1 correlated strongly with Foxp3+/CD4+ ratio. Taken together, increase of PD-1+ expression is associated with accumulation of Foxp3+ and CD8+ TILs. These findings intimate that PD-1/PD-L1 axis might shape the immune infiltration suggesting a modulation of the immune response and subsequent tumor growth in intracranial germinoma. Anti-PD-1 and anti-PD-L1 are potential immune therapeutic strategies in intracranial germinoma.

## Introduction

Central nervous system (CNS) germ cell tumors (GCTs) are common in Asia, where they account for 8–15% of all CNS tumors of childhood, compared to 3–4% in the United States [1–3]. Intracranial germinoma is the most common type of CNS GCT, accounting for up to two-thirds of all intracranial GCTs [2, 4]. Intracranial germinomas develop mostly in children, with a strong predilection for the young adult population [1–3]. Surgery followed by radiotherapy plus chemotherapy has achieved excellent survival outcomes for patients with intracranial germinomas [5, 6]. In about 10–20% of patients, however, the tumor recurs 10 years after first-line treatment [6]. Furthermore, since the majority of patients with this disease are children and adolescents, a large irradiated volume or high radiation dose results in late adverse effects such as growth disturbances or brain dysfunction[7]. Thus, with this highly curable disease, alternative therapeutic strategies for treating refractory tumors, preventing avoidable morbidity and maintaining quality of life have become the main goals of current pediatric oncological efforts.

Programmed cell death 1 (PD-1) is a receptor in the CD28 family, and plays an important role in immune tolerance and immune escape for a variety of tumor cells [8, 9]. One major ligand of PD-1 is programmed death ligand 1 (PD-L1). The PD-1/PD-L1 axis attenuates anti-tumor immune system as immune checkpoint [10]. The effector functions of T lymphocytes expressing PD-1 in the tumor microenvironment could be down-regulated upon activation by PD-L1, which is frequently expressed on tumor cells [11]. Immune checkpoint inhibitors have recently constituted a novel class of treatment that target ligands and receptors [12].

Large tumor cells of intracranial germinoma are frequently accompanied by an abundance of tumor-infiltrating lymphocytes (TILs), which demonstrate a characteristic histology known as the “two-cell pattern” [13]. It is no exaggeration to say that the genesis, development and survival of tumor cells depend on effective immune escape mechanisms. Recent research has revealed that immune escape is a complex process that includes tissue isolation, molecular simulation and immune suppression [11, 14]. One of the major components of this system is an immune checkpoint signal, the PD-1/PD-L1 axis [11]. However, expressions of PD-1 and PD-L1 as well as anti-tumor immune reactions remain unclear in intracranial germinoma.

This study examined expressions of PD-1 and PD-L1 in intracranial germinoma and characterized the subtypes of TILs showing surface antigens such as CD3+, CD8+, CD4+, and Foxp3+ using a quantitative evaluation method. Statistical analyses were performed to evaluate correlations among expressions of PD-1/PD-L1 axis and subtypes of TILs in intracranial germinoma.

## Materials and methods

### Patient selection and sample collection

We evaluated formalin-fixed, paraffin-embedded (FFPE) tumor specimens from patients with intracranial germinoma who were treated in Kyoto University Hospital between 2002 and 2016. We excluded two cases in which specimens were accompanied with large areas of necrosis and only small numbers of tumor cells were left. A total of 24 patients of surgically resected intracranial germinoma consisting of 22 primary and 3 recurrent tumors were recruited for this analysis. Among these, a pair of primary and recurrent tumors were from the same patient experienced recurrence, who underwent surgical resection twice in our institute. Clinical and pathological information was retrieved from the medical records and telephone interviews, including complete medical history, physical examination, magnetic resonance imaging (MRI) of the brain, and survival. After surgery, all patients received radiotherapy and chemotherapy. Three patients experienced recurrence, and one of those patients died. Demographic data and tumor characteristics are indicated in **Table 1**. Progression-free survival time was defined as the duration between initial treatment and identification of recurrence. Overall survival time was defined as the duration between initial treatment and date of death.

**Table 1:** Patient characteristics.

Case no.	Age (y)	Sex	Initial or recurrent	Lesion site	Multiple lesion	Serum β-hCG (nIU/mL)	Serum AFP (ng/mL)	Recurrence (months)	Overall survival (months)
1	15	M	Initial	Basal ganglia	+	0.95	< 3.0	—	189
2	27	M	Initial	Neurohypophysial	—	65	< 3.0	—	135
3	27	M	Initial	Pineal gland	—	< 0.5	< 3.0	—	153
4	21	F	Initial	Neurohypophysial	—	< 0.5	< 3.0	—	135
5	33	M	Initial	Pineal gland/ Neurohypophysial	+	< 0.5	1.9	—	121
6	23	M	Initial	Neurohypophysial	—	22.42	2.6	—	91
7	10	F	Initial	Neurohypophysial	—	< 0.5	3.2	—	89
8	10	F	Initial	Pineal gland/ Neurohypophysial	+	< 0.5	2.2	—	105
9	13	F	Initial	Pineal gland/ Neurohypophysial	+	< 0.5	1.0	—	101
10	22	M	Initial	Pineal gland	—	865.82	4.9	—	99
11	27	M	Initial	Pineal gland	—	< 0.5	1.4	—	99
12	22	M	Initial	Pineal gland	—	< 0.5	0.8	—	99
13	19	M	Initial	Pineal gland	—	2.02	< 0.6	—	89
14	14	F	Recurrent	Neurohypophysial	—	NA	NA	+ (276)	378
15	19	M	Recurrent	Basal ganglia/ Neurohypophysial	+	< 0.5	1.1	+ (154)	242 (died)
16*	12	F	Initial	Pineal gland	—	754.33	1.8	+ (45)	72
17	12	F	Initial	Pineal gland / Neurohypophysial	+	0.89	2.1	—	59
18	11	M	Initial	Basal ganglia	—	5.09	1.4	—	56
19	24	M	Initial	Pineal gland / Neurohypophysial	+	17.29	0.7	—	41
20	11	M	Initial	Neurohypophysial	—	3955.49	5.6	—	36
21	11	F	Initial	Neurohypophysial	—	16.31	1.7	—	29
22*	12	F	Recurrent	Pineal gland	—	0.58	1.8	+ (45)	72
23	19	M	Initial	Neurohypophysial	—	635.29	0.6	—	26
24	14	M	Initial	Basal ganglia	+	40.48	< 0.6	—	17
25	21	M	Initial	Pineal gland	—	< 0.5	< 0.6	—	3

Twenty-five consecutive intracranial germinoma patients were reviewed, including 22 primary cases and 3 recurrent cases. These included a pair of primary and recurrent specimens from the same patient, who had received surgical treatment twice at our institute. Abbreviations: β-hCG, β-human chorionic gonadotropin; AFP, alpha fetoprotein; NA, not applicable. * Case 16 and Case 22 represent the pair of primary and recurrent cases from the same patient, respectively.

This retrospective study was conducted in accordance with the Declaration of Helsinki, with approval from the ethics committee at Kyoto University Hospital. Written consent to use stored specimens was obtained from all living patients or their legal surrogates.

### Immunohistochemistry and immunohistochemical assessment

Five-micrometer thickness of FFPE tissue sections were baked at 60°C for 30 min, deparaffinized in xylene, and rehydrated in graded concentrations of ethanol. Heat-induced antigen retrieval was carried out in Tris-ethylenediaminetetraacetic acid buffer (pH 9.0) by microwaves, then endogenous peroxidase activity was quenched by incubating in 3% hydrogen peroxide at 37°C for 30 min. Monoclonal primary antibodies (anti-PD-1, 1/100, #329911, Biolegend, San Diego, CA; anti-PD-L1 [8–28], 1/100, ab205921, Abcam, Cambridge, UK; anti-CD3, 1/200, ab16669, Abcam; anti-CD4, 1/100, ab133616, Abcam; anti-CD8, 1/100, ab17147, Abcam; anti-Foxp3, 1/50, ab22510, Abcam; anti-OCT4, 1/100, ab181557, Abcam) were applied at 4°C overnight, then rewarming was performed at 37°C for 30 min, followed by incubation with biotinylated secondary antibodies (EnVision™+ Dual Link System-HRP; Dako, Santa Clara, CA) at 37°C for 30 min. To observe specific antibody localization, 3,3'-diaminobenzidine (DAB) (ImmPACT DAB Peroxidase Vector®; Vector Laboratories, Burlingame, CA) was used as the substrate and hematoxylin was used as a nuclear counterstain. Human placental tissue served as a positive control for PD-1 and PD-L1 antibodies, while human tonsil tissues served as positive control for CD3, CD4, CD8, and Foxp3 antibodies. For co-staining of PD-L1 and OCT4, 2 different peroxidase substrates were used (DAB for OCT4 and VECTOR® SG [Vector laboratories, Burlingame, CA] for PD-L1) and no counterstain was performed. For negative controls, slides were incubated with PBS in replacement of primary antibodies. Sections were examined and scored by two independent observers who were blinded to the clinicopathological background of patient samples. Sections for which evaluations differed were re-evaluated simultaneously by observers using a double-headed microscope and a consensus opinion was reached. All samples were stained at the same time.

### Evaluation of immunohistochemical staining for PD-1 and PD-L1 expressions

**Fig 1:**
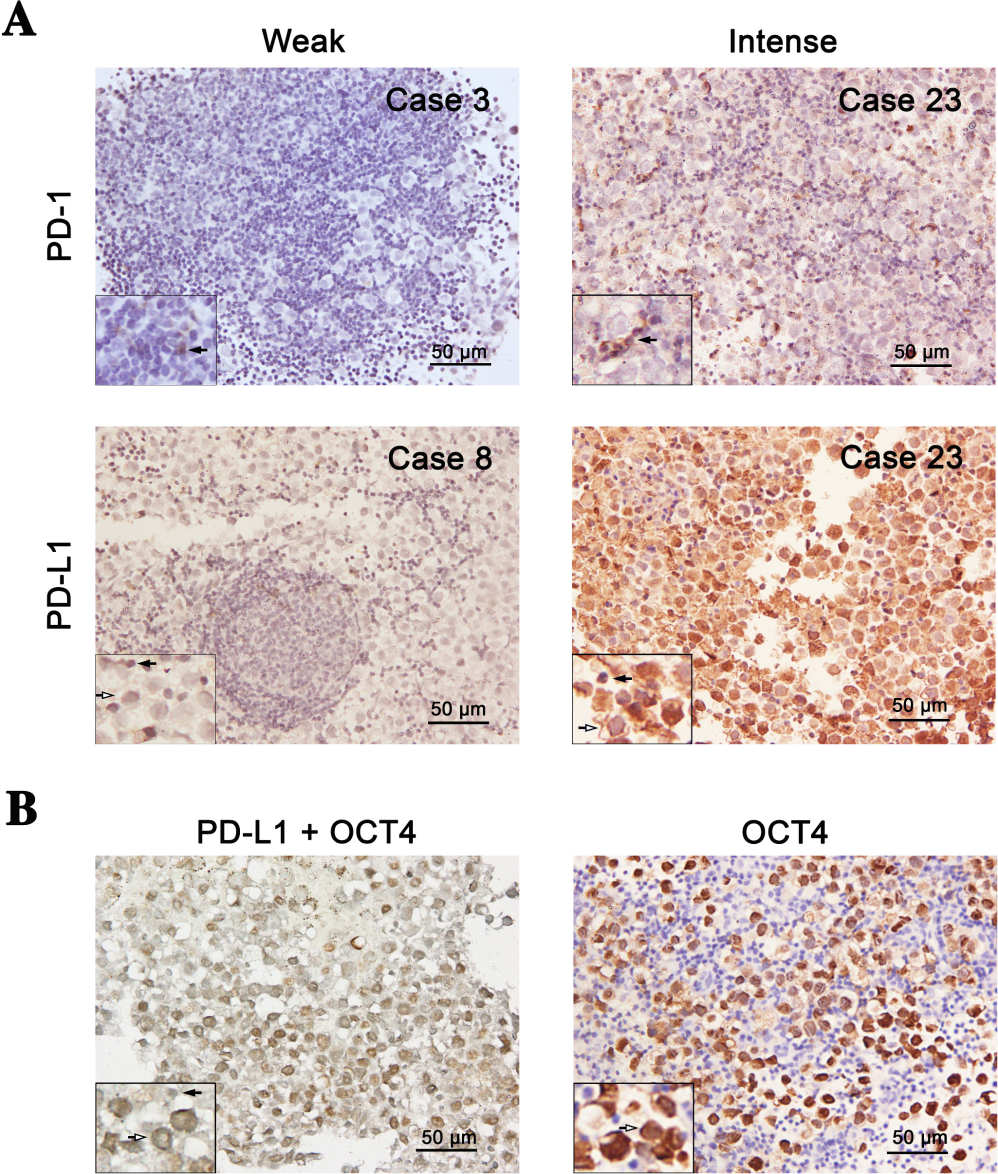
Representative weak or intensive immunohistochemical staining for PD-1 and PD-L1 expression in intracranial germinomas. (A) Variable intensities of PD-L1 expression in cancer cells and PD-1 in tumor-infiltrating lymphocytes were detected in intracranial germinomas. Representative images show weak and high expressions of PD-1 and PD-L1. (B) Representative images of co-staining of PD-L1 (blue-grey) and OCT4 (brown) and single staining of OCT4. Resected tumor tissues were immunostained with monoclonal anti-PD-1 (329911, Biolegend), anti-PD-L1 (ab210931, Abcam) and OCT4 (ab181557, abcam). Original magnification: ×400.

PD-1 and PD-L1 expressions were evaluated by calculating total immunostaining score (TIS) as the product of proportion score (PS) and intensity score (IS) [15]. PS represents the estimated fraction of positively stained cells of tumor cells (PD-L1) or TILs (PD-1), respectively (0, 0–5%; 1, 5–10%; 2, 10–25%; 3, 25–50%; 4, >50%). IS represents the estimated staining intensity as compared with control cells (0, no staining; 1, weak; 2, moderate; 3, intense) (Fig 1). TIS (TIS = PS × IS) ranges from 1 to 12, with only nine possible values (0, 1, 2, 3, 4, 6, 8, 9, and 12). Furthermore, we defined four subgroups: no expression, TIS 0; weak expression, TIS 1–4; moderate expression, TIS 6–8; and intense expression, TIS 9–12. Expressions of PD-1 and PD-L1 were analyzed as a dichotomous covariate in further statistical analyses, comparing high expression (TIS > 4) and low expression (TIS ≤ 4). For each sample, PD-1 expression on TILs and PD-L1 expression on tumors were distinguished depending on the morphology of the cells and the germinoma cell marker OCT4 expression [16]. A 5% cut-off value was applied for PD-1 and PD-L1 positivity, as proposed for non-small cell lung cancer and testicular germ cell tumors [17].

### Quantitative valuation of TILs

Quantification of TILs was undertaken by counting immunopositive cells in serial sections stained for lymphocyte differentiation antigens with the use of photomicrographs. CD3+, CD4+, CD8+, and Foxp3+ TILs were evaluated: 5 tumor areas with abundant TILs in a 400× magnification (high-power fields [HPF]) were pictured with the use of cellSens Standard 1.9 (Olympus, Tokyo, Japan). Enumeration of TIL were carried out using Qupath software, following the instruction on the official website (https://qupath.github.io/) [18]. TIL density was calculated as the mean number of counted cell numbers divided by field area (350 μm × 260 μm).

### Statistical analysis

Spearman correlation was applied to analyze correlations between two ordinal parameters. Unpaired Welch’s t test was used to compare group differences, as appropriate. A linear regression model was fitted for trend analysis. A two-tailed significance level *P* < 0.05 was applied. All statistical analyses were performed with Statistical Package for the Social Sciences (SPSS) version 20.0 software (SPSS, Chicago, IL).

## Results

### Highly frequent expressions of PD-1 and PD-L1 in intracranial germinomas

We found prominent PD-1 and PD-L1 expressions to variable extents in primary intracranial germinoma tissues. Representative immunohistochemical images for PD-1 and PD-L1 in intracranial germinoma are shown in Fig 1. In these tissues, all markers showed membrane-accentuated expression, also often accompanied by cytoplasmic expression. To visualize tumor cells, germinoma marker OCT4 was co-stained with PD-L1 in all cases [16]. In 25 tumor tissues, including all recurrent samples, expression of PD-1 in TILs was identified in 96% (24/25) and PD-L1 in tumor cells was identified in 92% (23/25). High expression of PD-1 (TIS > 4) in TILs and PD-L1 in tumor cells were detected in 76% (19/25) and 88% (22/25), respectively. In addition, PD-1 expression was not related to PD-L1 expression in the current cohort (*P* = 0.697).

### Quantifications and correlations of TIL subtypes in intracranial germinomas

As all specimens were FFPE tissues and most were biopsy samples, TILs were impossible to quantify with fluorescence-activated cell sorting techniques. Instead of classifying TILs into subgroups using semiquantitatively evaluation criteria [19], we quantified TIL density using the mean counts of immunopositive cells divided by area (350 μm × 260 μm, 0.091 mm^2^) under HPF.

**Fig 2:**
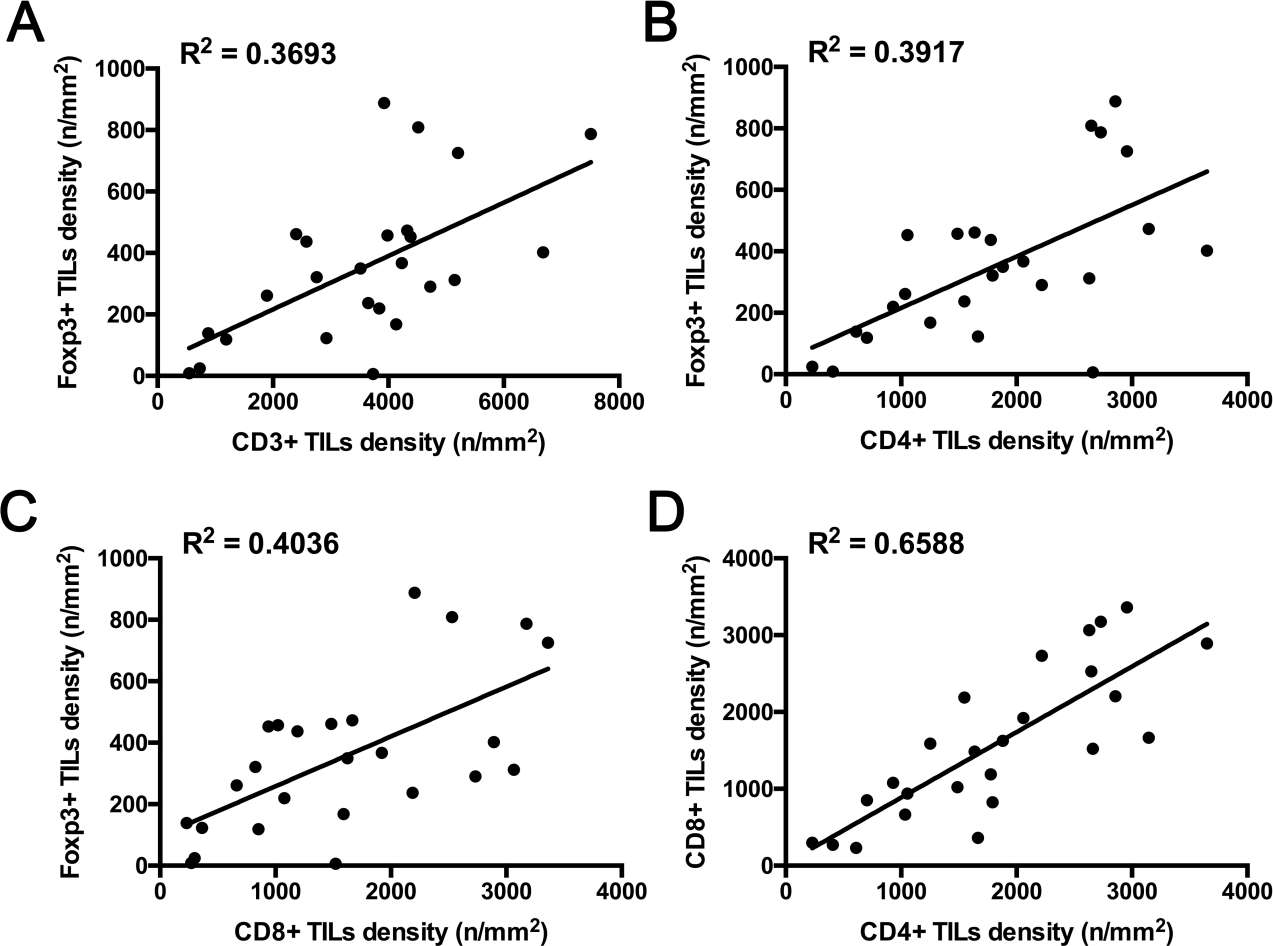
Correlation between densities of different tumor-infiltrating lymphocyte subtypes. Linear regression models reveal associations between Foxp3+ and CD3+ (A: P = 0.001), Foxp3+ and CD4+ (B: *P* < 0.001), Fox3+ and CD8+ (C: *P* < 0.001), and CD4+ and CD8+ (D: *P* < 0.001) TIL densities.

Widespread TILs of variable density with perivascular and dispersed foci were identified in intracranial germinomas. In detail, CD3+, CD4+, CD8+, and Foxp3+ TIL densities were (3577 ± 1724)/mm^2^ (range: 547–7510/mm^2^), (1823 ± 921)/mm^2^ (range: 231–3651/mm^2^), (1587 ± 968)/mm^2^ (range: 231–3360/mm^2^), and (344 ± 240)/mm^2^ (range: 9–888/mm^2^), respectively. Among TIL subtypes, Foxp3+ TIL density was associated with CD3+ (R^2^ = 0.3693, *P* = 0.001), CD4+ (R^2^ = 0.3917, *P* < 0.001), and CD8+ TIL density (R^2^ = 0.4036, *P* < 0.001), respectively. CD4+ TIL density correlated with CD8+ TIL density (R^2^ = 0.6588, *P* < 0.001). Linear regression models are shown in Fig 2.

### Associations between expression of PD-1 or PD-L1 and TIL subtype densities

**Fig 3:**
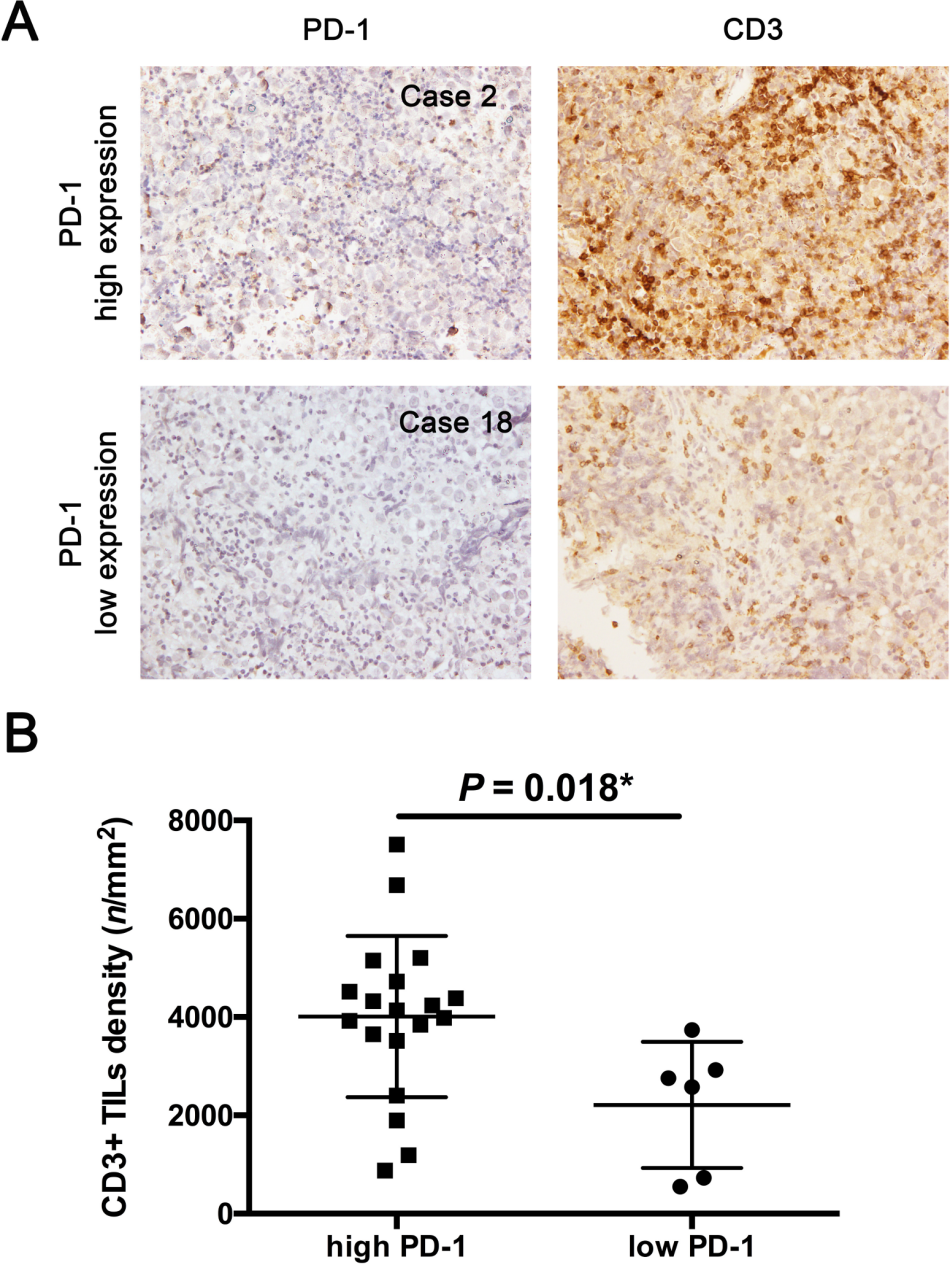
Expression of PD-1 correlated with total TIL density (CD3+). (A) Representative immunohistochemical images of PD-1 and CD3 proteins show that CD3+ TIL density was higher in patients with high expression of PD-1 (Case 2) than in the patient with low expression of PD-1 (Case 18). Figures were taken from the same areas of tissue, respectively. (B) Unpaired Welch’s t testing reveals that CD3+ TIL density was higher in primary germinoma cases showing high expression of PD-1 (*P* = 0.018). Black arrow, TIL; white arrow, cancer cell. Original magnification: ×400.

**Fig 4:**
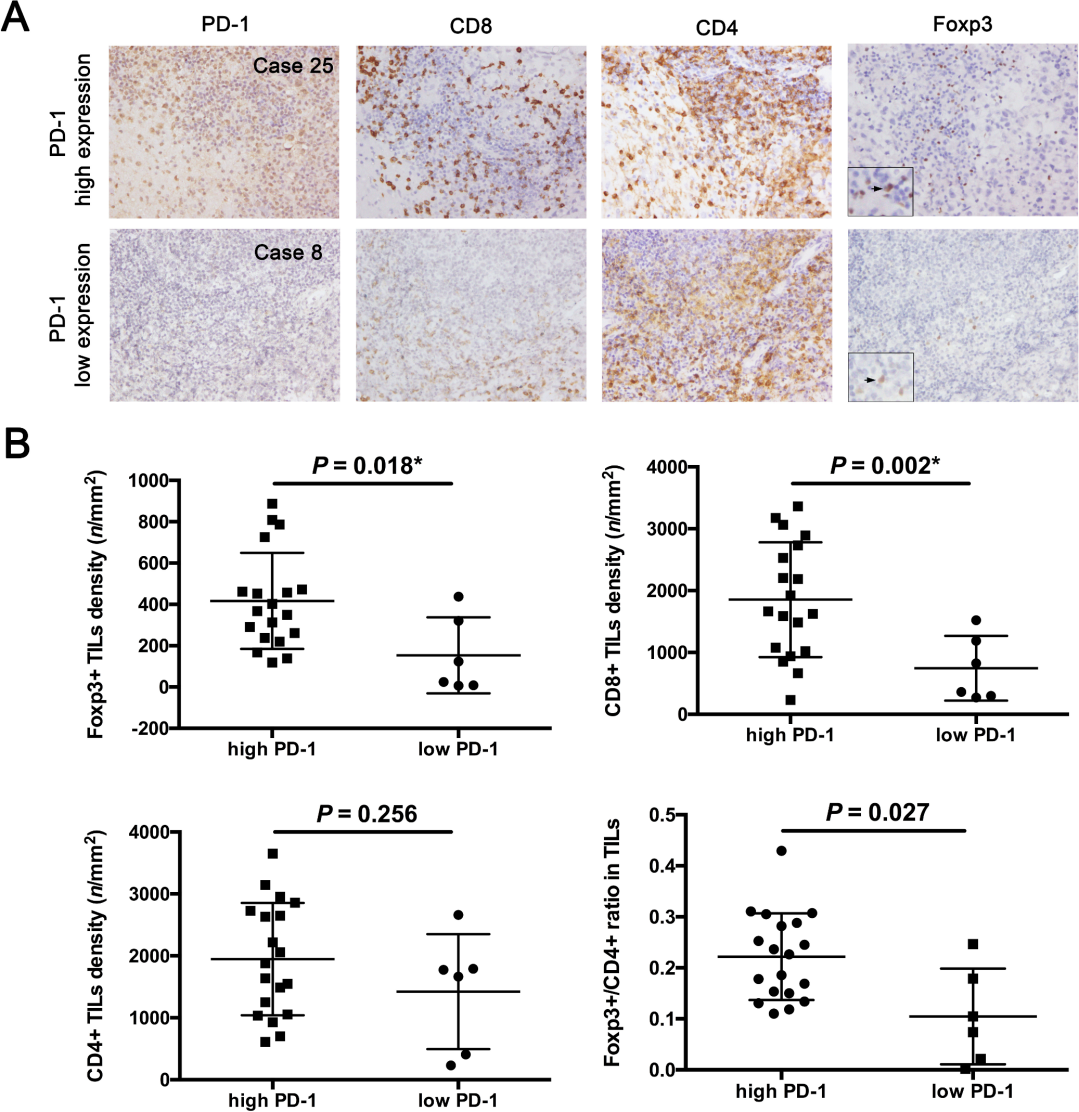
Correlations between PD-1 expression in lymphocytes and TIL subtype densities in primary intracranial germinoma. (A) Representative immunohistochemical images for PD-1, CD4, CD8, and Foxp3 staining in two patients with PD-1 high expression (TIS > 4; Case 25) and PD-1 low expression (TIS ≤ 4; Case 8). All representative photographs were taken in the same area of each tissue. Compared with Case 8, Case 25 showed higher PD-1 expression in TIL, higher CD8+TILs and Foxp3+ TILs. (B) Compared with low expression of PD-1, high expression of PD-1 correlated with higher Foxp3+ TIL density, CD8+ TIL density, and Foxp3+/CD4+ ratio, but not CD4+ TIL density. All P values are according to the unpaired Welch’s t test.

In 22 initial germinoma cases, CD3+ TIL density was significantly higher among patients with high expression of PD-1 than in those with low expression of PD-1 (4008 ± 376.3/mm^2^ versus 2212 ± 523.6/mm^2^, *t* = 2.785, *P* = 0.018) (Fig 3A and 3B). We found higher Foxp3+ density (416.7 ± 53.4/mm^2^ versus 153.5 ± 75.0/mm^2^, *t* = 2.860, *P* = 0.016) and higher CD8+ TIL density (1853 ± 212.9/mm^2^ versus 744.7 ± 214.2/mm^2^, *t* = 3.671, *P* = 0.002) in patients with high expression of PD-1 than in those with low expression of PD-1 (Fig 4). However, no significant difference in CD4+ TIL density was evident between patients with high and low expressions of PD-1 (1949 ± 208.0/mm^2^ versus 1422 ± 378.7/mm^2^, *t* = 1.220, *P* = 0.256) (Fig 4). Furthermore, Foxp3+/CD4+ ratio was higher in patients with high expression of PD-1 than in those with low expression of PD-1 (0.222 ± 0.019 versus 0.105 ± 0.038, *t* = 2.727, *P* = 0.027) (Fig 4). In the 2 PD-L1 negative cases (case 3 and 8), the CD8+ TILs (1520.9/mm^3^ and 362.6/mm^3^, respectively) were all smaller than the mean of CD8+ TILs (1587/mm^3^) in total. No correlation was seen between PD-L1 expression in tumor cells and TIL subtypes (**Table 2**).

**Table 2:** PD-1 and PD-L1 expressions and TILs density in 25 germinoma tissues.

Case no.	PD-1 expression in TIL	PD-L1 expression in cancer cell	TIL density (*n*/mm^3^)			
			CD3+	CD4+	CD8+	Foxp3+
1	+++	+++	3984.6	1487.9	1022.0	402.2
2	+++	++	4725.3	2219.8	2731.9	123.1
3	+	—	3736.3	2661.5	1520.9	219.8
4	+++	++	4382.4	1055.0	938.5	167.6
5	++	++	876.9	611.0	230.8	237.4
6	+++	+++	4235.2	2059.3	1920.9	312.1
7	+++	+++	6683.5	3650.5	2892.3	786.8
8	+	—	2925.3	1665.9	362.6	887.9
9	+++	+++	3841.8	929.7	1076.9	261.5
10	+++	++	4136.3	1252.7	1589.0	808.8
11	+++	+	3650.5	1547.5	2186.8	8.8
12	+++	++	5149.5	2630.8	3065.9	437.4
13	++	+++	7509.9	2729.7	3173.6	472.5
14#	+++	+++	3925.3	2857.1	2206.6	320.9
15#	+++	+++	1896.7	1035.2	663.7	461.5
16*	++	+++	4518.7	2648.4	2529.7	349.5
17	—	+++	546.7	408.8	270.3	24.2
18	+	+++	2578.0	1775.8	1189.0	118.7
19	+++	+++	4325.3	3145.1	1665.9	725.3
20	+	+++	2756.0	1791.2	826.4	402.2
21	+++	+++	2402.2	1637.4	1483.5	123.1
22*	+++	+++	3519.2	1881.3	1624.2	219.8
23	+	+++	731.9	230.8	298.9	167.6
24	+++	+++	1189.0	703.3	852.7	237.4
25	+++	+++	5206.6	2956.0	3360.4	312.1

* Case 16 and case 22 are a pair of initial and recurrent cases of the same patient, respectively.

# The patients experienced pre-operative radiochemotherapy and recurrence.

### TIL subtype densities in recurrent germinomas

**Fig 5:**
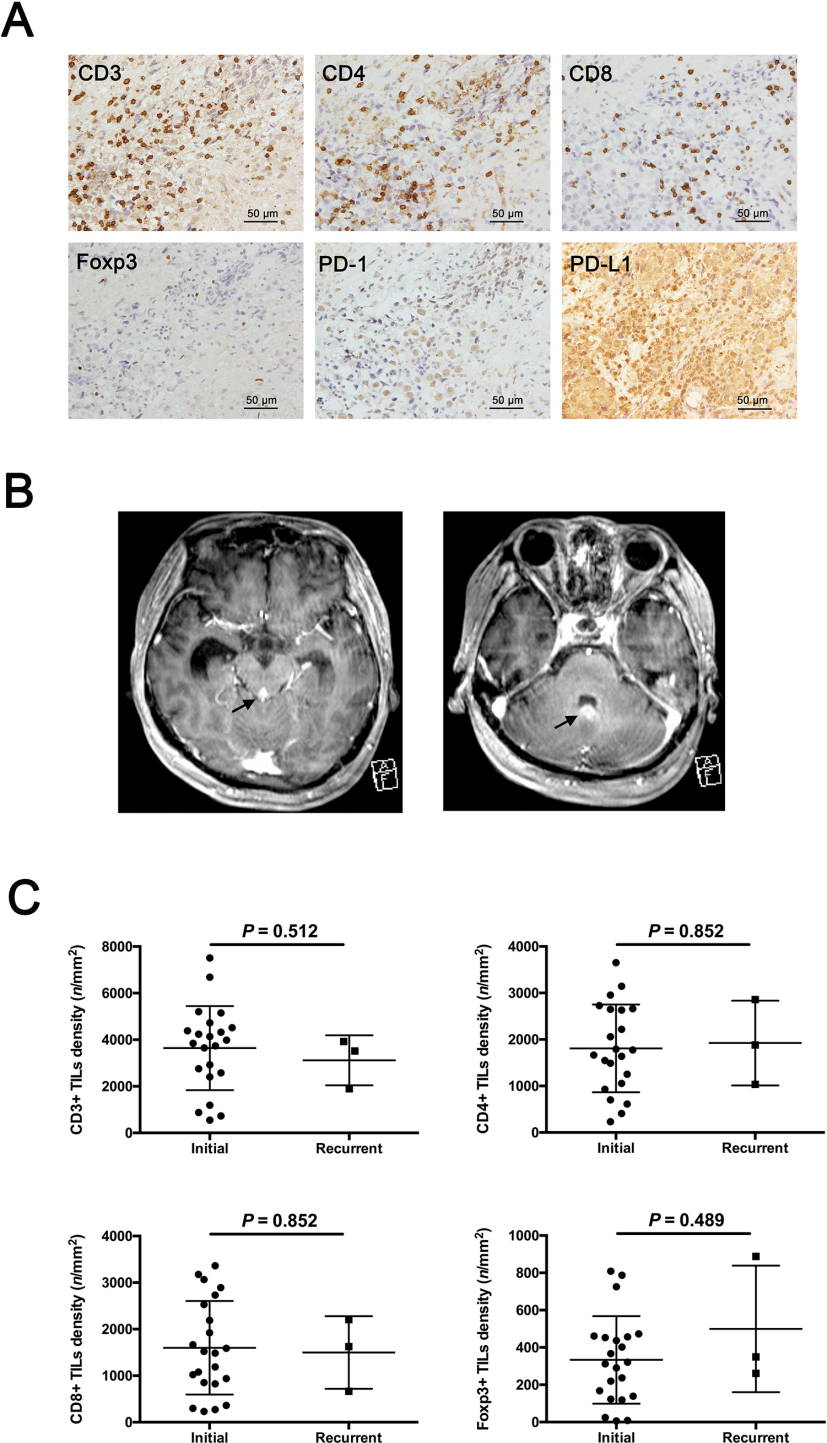
TIL subtype densities in initial and recurrent germinomas. (A) Representative immunohistochemical images for CD3, CD4, CD8, Foxp3, PD-1, and PD-L1 in the patient (Case 15) who died from the disease (progression-free time, 154 months; overall survival, 242 months). High CD3+, CD4+, CD8+, and Foxp3+ TIL densities were detected. Moderate membranous PD-1 expression (TIS = 8) and intense membranous PD-L1 expression (TIS = 12) in tumor cells were detected. Photographs were taken under high-power fields (original magnification ×400). (B) Gadolinium-enhanced MRI shows disseminated tumor masses (black arrow). (C) Comparison of TIL subtype densities between initial and recurrent germinoma tissues. No difference of CD3+, CD4+, CD8+, and Foxp3+ TIL densities was found between initial and recurrent cases. All P values were calculated according to the un-paired Welch’s t test.

During follow-up of 6 months to 31.5 years, 3 patients (Cases 14–16) were refractory to treatment and experienced recurrence, and Case 15 eventually died from the tumor. Moderate and high expressions of PD-1 and PD-L1 were observed in these 3 recurrent patients. In the dead case (Case 15), relatively high CD3+, CD4+, CD8+, and Foxp3+ TIL densities were observed (Fig 5A). MRI showed dissemination of germinoma at the final recurrence (Fig 5B). In all recurrent cases, high expressions of PD-1 and PD-L1 were detected. No difference of CD3+, CD4+, CD8+, and Foxp3+ TIL densities were found between initial and recurrent germinoma (Fig 5C).

### Associations between clinicopathological parameters and PD-1, PD-L1, expression and TIL subtype densities

We performed univariate analysis with expressions of PD-1 and PD-L1 and clinicopathological parameters in the 22 primary cases. Detailed results are shown in **Table 3**. We identified correlations between high expression of PD-1 in TILs and high serum β-hCG levels (cut-off, 100 mIU/mL; *n* = 18 for β-hCG < 100 mIU/mL and *n* = 4 for β-hCG ≥ 100 mIU/mL; *P* = 0.040). PD-L1 expression was stronger in patients < 18 years old (*P* = 0.018). No other associations were found between other clinicopathological parameters and expressions of PD-1 or PD-L1.

**Table 3:** Correlation between PD-1 and PD-L1 expression and clinical parameters of 22 initial intracranial patients.

	*n*	PD1					PD-L1				
		TILs				*P* value	Tumor cells				*P* value
		—	+	++	+++		—	+	++	+++	
Total	22	1	5	3	13		2	1	6	13	
Age						0.308					0.018
< 18 y	10	1	3	1	5		1	0	0	9	
≥ 18 y	12	0	2	2	8		1	1	6	4	
Gender						0.788					0.561
Male	15	0	4	2	9		1	1	5	8	
Female	7	1	1	1	4		1	0	1	5	
β-hCG (nIU/mL)						0.040					0.424
< 100	18	0	3	3	12		2	1	5	10	
≥ 100	4	1	2	0	1		0	0	1	3	
AFP (ng/mL)						0.876					0.674
< 3	19	1	4	3	11		2	1	5	11	
≥ 3	3	0	1	0	2		0	0	1	2	
Lesion site						0.808					0.675
Neurohypophysial	8	1	2	0	5		0	0	2	6	
Pineal gland	11	0	2	3	6		2	1	4	4	
Basal ganglia	3	0	1	0	2		0	0	0	3	
Multifocal lesions						0.788					0.877
No	15	0	4	2	9		1	1	4	9	
Yes	7	1	1	1	4		1	0	2	4	

Abbreviations: *βhCG*, beta human chorionic gonadotropin β subunit; *AFP* alpha fetoprotein.

## Discussion

The present study systematically investigated expressions of PD-1 and PD-L1 in intracranial germinoma. We identified frequent expression of PD-L1 (23/25, 92%) in germinomatous components as well as frequent expression of PD-1 in TILs (24/25, 96%). Aoki et al reported that PD-L1 was expressed on immune cell containing tumor but not on tumor cells in a study of 7 pediatric intracranial germinomas (median age, 2 years; range: 0 months-16 years) [20]. Consistent with the current study, Fankhauser et al. reported frequent expression of PD-L1 in testicular seminoma (73%), a kind of non-brain germ cell tumor with identical histology to intracranial germinoma [17]. Intracranial germinoma is generally considered as the counterpart of “seminoma” in the testis and “dysgerminoma” in the ovary, constituting the neoplastic counterpart of the primordial germ cell, just as embryonal carcinoma is the neoplastic counterpart of totipotent embryonal stem cells and teratoma the neoplastic counterpart of the derivatives of the three embryonic germ layers [21]. However, little is known of the exact genetic correlation between germinoma and seminoma. Cierna et al. reported similar PD-L1 expression in testicular seminoma with a frequency of 76% [22]. Frequency of PD-L1 expression in our cohort was comparable with that in other brain tumors such as glioblastoma (88.0–100%) [14, 23], anaplastic astrocytoma (71.4%) [14] and medulloblastoma (56.3%) [8]. In many types of non-brain tumor, frequent tumoral expression of PD-L1 was also reported with tumors such as ovarian carcinoma (86.7%) [24], non-small cell lung cancer (53%) [25], and colorectal cancer (36%) [26], renal cell carcinoma (32%) [27] and melanoma [28]. The discrepancies between PD-L1 expression in germinomas among studies might be attributable to the use of primary antibodies from different manufacturers or different immunoreactivities between the manual operation in our study and the automated staining system used in the study by Aoki et al. Taken together, previous investigations into the same category of pathology and our study of 24 patients offer positive confirmation of the frequent expression of PD-L1 in intracranial germinoma.

The histology of intracranial germinoma is characteristic, exhibiting numerous TILs among large tumor cells, as the so-called “two-cell pattern”, suggesting a peculiar host-immune response to the tumor. In our cohort, numerous TILs were also found in all 25 samples of intracranial germinoma tissue (Fig 2). The majority of TILs have been reported to be T-lymphocytes. In 1979, Neuwelt and Smith reported T-lymphocyte membrane marker as present in 51% of small cells in germinoma tissues [29]. Saito et al. also reported that 70–80% of TILs were T lymphocytes, infiltrating either diffusely or in clusters, whereas 20–30% of TILs were B lymphocytes that tended to cluster in tumor tissues [30]. TILs have recently received close attention in terms of anti-tumor immune function. Since functions of immune checkpoint signals, such as the PD-1/PD-L1 axis, were discovered in cancer, most studies have focused on correlations between PD-1, PD-L1 and TILs using semiquantitatively analyses, but limited studies have reported the details of infiltrating T-lymphocyte subtypes, particularly in central nervous system tumors. Berghoff et al. reported that, in glioblastoma, PD-1+ TIL density correlated positively with CD3+ TIL density and CD8+ TIL density [23]. Our previous study revealed that high expression of PD-L1 was associated with reduced infiltration of CD8+ T cells and poor prognosis in medulloblastoma [8]. The current study analyzed the density of TIL subtypes with quantified data. As a result, expression of PD-1 in TILs correlated positively with CD8+ TIL and CD3+ TIL densities. In addition, expression levels of PD-1 in TILs did not influence CD4+ TIL density. However, the underlying mechanisms require further research for interpretation. Interactions between immune and tumor cells play cardinal roles during malignant progression [31]. Furthermore, dysfunction of the anti-tumor immune system may result in the progression of germinoma to the development of symptoms.

Intracranial germinomas arise mostly in the neurohypophysis (30.1%) and pineal regions (51%), but infrequently in the basal ganglia (3.3%), cerebellopontine angle (2.6%), lateral ventricle (2%), cerebellum (2%) and multiple sites (8.5%) [32]. Germinoma cells actually show the malignant characteristic of infiltration into brain parenchyma, especially around the subventricular regions, and display recurrence at different areas and dissemination into the CSF space. Intracranial germinomas are highly radiosensitive and potentially curable using radiotherapy alone [33, 34]. As well documented in the literature, CNS radiation for pediatric patients leads to dysfunctions in growth [35], the endocrine milieu [36], and neurocognition [37]. A reduced dose of radiotherapy to the whole ventricular area with chemotherapy has been introduced into treatment and has yielded promising therapeutic responses [6, 38, 39]. However, the side effects of radiotherapy remain inevitable, even when dose and target volumes have been reduced. Meanwhile, clinical trials to substitute radiation with chemotherapy have resulted in increased relapse rates and inferior outcomes, and chemotherapy alone is thus currently not an acceptable treatment option [40, 41]. Despite these efforts to establish reduced radiotherapy protocols in combination with chemotherapy, 10–20% of intracranial germinomas are bound to recur after first-line treatment and no standard second-line treatments have been defined [6, 42–44]. To conquer radiation-related side effects as well as recurrent tumors, a major task is to develop a novel therapeutic option for germinoma. Our results suggest immune checkpoint therapy as one possible option. Antibodies targeting checkpoint molecules such as PD-1 have already shown therapeutic effects on non-small cell lung cancer and melanoma, and PD-L1 expression exhibited a positive correlation with response to PD-1 inhibition in those tumors [45, 46]. Preliminary efficacy of PD-1 antibodies was reported in seven patients with relapse of extracranial GCT after high-dose chemotherapy and stem cell transplantation [47]. Three of those patients received PD-1 antibodies for at least 6 months and long-term tumor response was achieved in two of the three patients, with tumor tissues showing highly positive results for PD-L1 staining. This report encourages examination of the efficacy of immune checkpoint therapy for intracranial germinoma. Given the existence of blood-brain barrier (BBB), the penetrance of PD-1 antibodies into CNS via BBB is not fully known. However, the BBB disruption in brain neoplasms could be observed with a marked heterogeneity [48]. In addition, radiotherapy, a standard therapeutic approach in intracranial germinomas, could further facilitate the penetrance of activated anti-tumor immune cells [49] and possibly the access of PD-1 antibodies. Actually, many clinical trials are ongoing to explore PD-1 in patients with primary (NCT02017717) or metastatic (NCT02320058) brain tumors. However, planning a suitable clinical trial is difficult, because intracranial germinoma is rare, and relapsed cases are even rarer. An international study for this context is thus desirable to develop novel approaches to the treatment of intracranial germinoma.

## Conclusion

In the present study, expressions of PD-1 and PD-L1 were detectable in 96% (24/25) and 92% (23/25) of germinoma tissues, respectively. PD-1 expression in lymphocytes was associated with Foxp3+ TIL density. Increases in PD-1+ TILs was associated with accumulation of CD8+ TILs, which might be caused by immune dysfunction. Induction of Foxp3+ TILs from CD4+TILs causes immune tolerance. These results intimate the breakdown of the tumor immune system that might be induced by PD-1/PD-L1 axis in intracranial germinoma, suggesting the potential efficacy of immune checkpoint therapy.
